# Supplementary material for: Development of an expected possession value model to analyse team attacking performances in rugby league
Source: PLoS One. 2021 Nov 12;16(11):e0259536. doi: 10.1371/journal.pone.0259536 (PMC8589207; doi:10.1371/journal.pone.0259536)

**S1 File**

In this supplemental data, all calculations for the determination of the four aggregated zone size models based on the zones’ match EPV are shown. All four sets of zones were generated by grouping together EPV-308 states based on their spatial similarity and match EPV obtained per fixture.

*Initial summation of column/row match EPV*

First, the match EPV (G_m_(s,t) for each match m) was calculated using Equation 2 from the manuscript. These values were summated at a column level to provide the column match EPV for fourteen 5m columns along the x-axis (Table 1). They were also summated at the row level to provide the row match EPV for twenty two 5m rows along the y-axis (Table 2). All individual match EPVs were added to a linear mixed model as specified in the manuscript (with column/row match EPV as fixed effects, team/fixture ID as random effects) so that means and 95% confidence intervals could be obtained. These values are shown in Tables 1 and 2.

| **Table 1:** Total Match EPV obtained per column per fixture. | |
| --- | --- |
| **Column (metres included)** | **Column Match EPV per fixture (95% Confidence Intervals)** |
| 1 (0-3m) | 1.41 (1.04 – 1.78) |
| 2 (4-8m) | 2.72 (2.38 – 3.06) |
| 3 (9-13m) | 4.52 (4.18 – 4.87) |
| 4 (14-18m) | 6.06 (5.72 – 6.40) |
| 5 (19-23m) | 7.10 (6.75 – 7.44) |
| 6 (24-28m) | 8.57 (8.23 – 8.92) |
| 7 (29-33m) | 12.78 (12.44 – 13.12) |
| 8 (34-38m) | 12.27 (11.92 – 12.61) |
| 9 (39-43m) | 9.72 (9.38 – 10.07) |
| 10 (44-48m) | 7.76 (7.41 – 8.10) |
| 11 (49-53m) | 5.71 (5.37 – 6.05) |
| 12 (54-58m) | 5.43 (5.09 – 5.78) |
| 13 (59-63m) | 2.55 (2.20 – 2.89) |
| 14 (64-68m) | 1.13 (0.76 – 1.50) |
| **N.B.:** Column metres are inclusive and measured from the left side of the pitch based on the journal figures. EPV values are reported in points. | |

| **Table 2:** Total Match EPV obtained per row per fixture. | |
| --- | --- |
| **Row (metres included)** | **Row Match EPV per fixture (95% Confidence Intervals)** |
| 1 (-10 to -5m) | 0.19 (-0.38 – 0.76) |
| 2 (-4 to 0m) | 0.45 (0.09 – 0.81) |
| 3 (1-5m) | 1.12 (0.86 – 1.38) |
| 4 (6-10m) | 2.12 (1.86 – 2.38) |
| 5 (11-15m) | 3.31 (3.05 – 3.57) |
| 6 (16-20m) | 3.21 (2.95 – 3.47) |
| 7 (21-25m) | 3.15 (2.89 – 3.41) |
| 8 (26-30m) | 3.22 (2.97 – 3.48) |
| 9 (31-35m) | 2.88 (2.62 – 3.14) |
| 10 (36-40m) | 3.00 (2.75 – 3.26) |
| 11 (41-45m) | 3.38 (3.12 – 3.64) |
| 12 (46-50m) | 3.03 (2.77 – 3.29) |
| 13 (51-55m) | 3.59 (3.33 – 3.85) |
| 14 (56-60m) | 4.16 (3.90 – 4.42) |
| 15 (61-65m) | 3.67 (3.42 – 3.93) |
| 16 (66-70m) | 4.03 (3.77 – 4.28) |
| 17 (71-75m) | 4.91 (4.65 – 5.17) |
| 18 (76-80m) | 5.29 (5.03 – 5.55) |
| 19 (81-85m) | 6.72 (6.46 – 6.98) |
| 20 (86-90m) | 9.12 (8.86 – 9.38) |
| 21 (91-95m) | 11.43 (11.17 – 11.68) |
| 22 (96-100m) | 6.52 (6.25 – 6.79) |
| **N.B.:** Row metres are inclusive and measured from team in possession’s try line (0m). EPV values are reported in points. | |

*Smoothing due to spatial similarity*

Visual inspection of Table 1 shows that the values follow a normal distribution, peaking in the middle and much smaller in wider areas. In order to ensure that teams who attack in wide areas could be evaluated equally, regardless of whether they attacked from the left or right side of the pitch, the column values were averaged symmetrically (i.e. the two widest columns (1 & 14) were averaged, the two most central columns (7 & 8) and all other pairs in between. The column match EPV for the resulting seven 10m wide columns are shown in Table 3.

Visual inspection of Table 2 showed a similar spatial trend whereby the row match EPV increased as the row moved closer to the opposition try line. To smooth these changes and remove any potential outliers (e.g. row 14 or 15, and row 22), the values were once again averaged into 10m rows. This resulted in eleven 10m rows, shown in Table 4.

These columns and rows formed the basis for the linear mixed model analysis outlined below.

| **Table 3:** Averaged column match EPVs. Columns averaged by visual inspection of data. | |
| --- | --- |
| **Column**  **(Columns included from Table 1)** | **Column Match EPV per fixture (95% Confidence Intervals)** |
| 1 (1,14) | 1.27 (0.97 – 1.57) |
| 2 (2,13) | 2.63 (2.35 – 2.92) |
| 3 (3,12) | 4.98 (4.70 – 5.26) |
| 4 (4,11) | 5.88 (5.60 – 6.17) |
| 5 (5,10) | 7.43 (7.14 – 7.71) |
| 6 (6,9) | 9.15 (8.87 – 9.43) |
| 7 (7,8) | 12.52 (12.24 – 12.81) |
| **N.B.:** EPV values are reported in points. | |

| **Table 4:** Averaged row match EPVs. Rows averaged due to visual inspection of data. | |
| --- | --- |
| **Row**  **(Rows included from Table 2)** | **Row Match EPV per fixture (95% Confidence Intervals)** |
| 1 (1,2) | 0.32 (-0.02 – 0.67) |
| 2 (3,4) | 1.62 (1.42 – 1.82) |
| 3 (5,6) | 3.26 (3.06 – 3.46) |
| 4 (7,8) | 3.19 (2.99 – 3.39) |
| 5 (9,10) | 2.94 (2.74 – 3.14) |
| 6 (11,12) | 3.20 (3.00 – 3.41) |
| 7 (13,14) | 3.87 (3.67 – 4.08) |
| 8 (15,16) | 3.85 (3.65 – 4.05) |
| 9 (17,18) | 5.10 (4.90 – 5.30) |
| 10 (19,20) | 7.92 (7.72 – 8.12) |
| 11 (21,22) | 8.97 (8.77 – 9.18) |
| **N.B.:** EPV values are reported in points. | |

*Linear mixed model analysis*

In separate linear mixed models (column/row match EPV as fixed effects; team/fixture ID as random effects), the row and column match EPVs were compared against a smallest effect size of interest (SESOI) of 0.5, 1.0 and 1.5 points per fixture respectively. Where a significant difference (P < 0.05) was present between two columns/rows, they were kept separate. When the difference was not significant, the two columns/rows were averaged together and compared to the next column/row. This analysis resulted in EPV-37 (SESOI = 0.5), EPV-19 (SESOI = 1.0), EPV-13 (SESOI = 1.5) and EPV-9 (SESOI = 2)..

*EPV-37 (SESOI = 0.5)*

An SESOI of 0.5 indicates that we are testing whether across 2 matches, a column/row would be at least 1 point more valuable than the compared column/row. Table 5 shows all the comparisons completed with an SESOI of 0.5 for the columns of the pitch. Table 6 outlines the subsequent columns used in EPV-37 and the metres included on the pitch. Table 7 provides the comparisons completed for all rows on the pitch against an SESOI of 0.5. Table 8 provides the final rows used in EPV-37 and the metres included on the pitch.

| **Table 5:** Statistical comparisons between columns at an SESOI of 0.5. Where numbers are separated by commas, they have been averaged. Difference and significance provide the mean difference (95% confidence intervals) between the columns and whether this was significant according to an SESOI of 0.5. Combine? Indicates whether the column values were averaged before comparing to the next column. Comparisons began at the outermost columns before progressing more centrally. | | | | |
| --- | --- | --- | --- | --- |
| **Columns included** | **Difference** | | **Significance** | **Combine?** |
| 2 - 1 | 1.36 (1.06 – 1.66) | | *P*  < 0.0001 | No |
| 3 - 2 | 2.35 (2.06 – 2.63) | | *P*  < 0.0001 | No |
| 4 - 3 | 0.91 (0.62 – 1.18 | | *P*  = 0.0025 | No |
| 5 - 4 | 1.54 (1.26 – 1.82) | | *P*  < 0.0001 | No |
| 6 - 5 | 1.72 (1.44 – 2.00) | | *P*  < 0.0001 | No |
| 7 - 6 | 3.38 (3.09 – 3.66) | | *P*  < 0.0001 | No |
| **Table 6:** Final columns for EPV-37 and the average column match EPV obtained per fixture across the 2019 Super League season | | | | |
| **Column**  **(metres included)** | | **Column Match EPV per fixture (95% Confidence Intervals)** | | |
| 1 (0-3, 64-68m) | | 1.27 (0.97 – 1.57) | | |
| 2 (4-8, 59-63m) | | 2.63 (2.35 – 2.92) | | |
| 3 (9-13, 54-58m) | | 4.98 (4.70 – 5.26) | | |
| 4 (14-18, 49-53m) | | 5.88 (5.60 – 6.17) | | |
| 5 (19-23, 44-48m) | | 7.43 (7.14 – 7.71) | | |
| 6 (24-28, 39-43m) | | 9.15 (8.87 – 9.43) | | |
| 7 (29-38m) | | 12.52 (12.24 – 12.81) | | |
| **N.B.:** Column metres are inclusive and measured from the left side of the pitch based on the journal figures. EPV values are reported in points. | | | | |

| **Table 7:** Statistical comparisons between rows at an SESOI of 0.5. Where numbers are separated by commas, they have been averaged. Difference and significance provide the mean difference (95% confidence intervals) between the rows and whether this was significant according to an SESOI of 0.5. Combine? Indicates whether the row values were averaged before comparing to the next row. Comparisons began inside the attacking teams try area before progressing up the pitch to the opposition try area. | | | |
| --- | --- | --- | --- |
| **Rows included** | **Difference** | **Significance** | **Combine?** |
| 2 - 1 | 1.30 (0.93 – 1.67) | *P*  < 0.0001 | No |
| 3 - 2 | 1.64 (1.41 – 1.87) | *P*  < 0.0001 | No |
| 4 - 3 | -0.07 (-0.30 – 0.16) | *P*  = 0.5542 | Yes |
| 5 - 3,4 | -0.28 (-0.48 – -0.08) | *P*  = 1.000 | Yes |
| 6 - 3,4,5 | 0.07 (-0.12 – 0.26) | *P*  = 1.000 | Yes |
| 7 - 3,4,5,6 | 0.73 (0.54 – 0.91) | *P*  = 0.0079 | No |
| 8 - 7 | -0.02 (-0.26 – 0.21) | *P* = 1.000 | Yes |
| 9 - 7,8 | 1.24 (1.04 – 1.44) | *P* < 0.0001 | No |
| 10 - 9 | 2.82 (2.59 – 3.05) | *P* < 0.0001 | No |
| 11-10 | 1.06 (0.82 – 1.29) | *P* < 0.0001 | No |

| **Table 8:** Final rows for EPV-37 and the average row match EPV obtained per fixture across the 2019 Super League season | |
| --- | --- |
| **Row**  **(metres included)** | **Total Row EPV per fixture (95% Confidence Intervals)** |
| 1 (-10 to 0m) | 0.32 (-0.03 – 0.67) |
| 2 (1 to 10m) | 1.62 (1.42 – 1.82) |
| 3 (11m to 50m) | 3.15 (3.00 – 3.30) |
| 4 (51m to 70m) | 3.86 (3.70 – 4.03) |
| 5 (71m to 80m) | 5.10 (4.90 – 5.30) |
| 6 (81m to 90m) | 7.92 (7.72 – 8.12) |
| 7 (91m to 100m) | 8.97 (8.77 – 9.18) |
| **N.B.:** Row metres are inclusive and measured from team in possession’s try line (0m). EPV values are reported in points. | |

After obtaining the columns and rows, a 49 zone model was created. However, due to the significantly reduced number of visits to all zones in rows 1 and 2, it was decided to average across them, forming a single zone for row 1 (-10m to 0m) and a single zone for row 2 (1m to 10m). The resulting EPV-37 is shown in Figure 2.

Having identified the zones based on statistical analysis of their match EPVs, the zone values were calculated by taking the weighted average of all the EPV-308 zones that they covered. For example, zone 1’s value was the weighted average of the 28 EPV-308 zones it covered. See Figure 1 in the manuscript for more details.

**Figure 1: EPV-37. Left: Blank zones; Right: zone values, coloured as per Figure 2 in manuscript.**


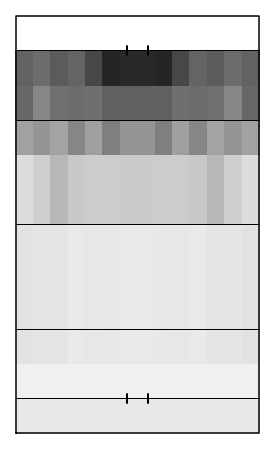

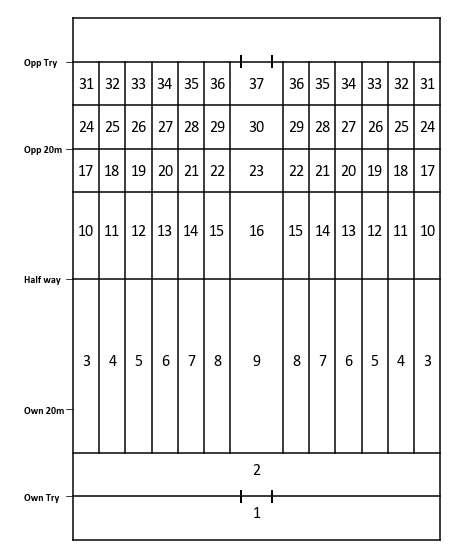


*EPV-19 (SESOI = 1.0)*

An SESOI of 1.0 indicates that we are testing whether a column/row is at least 1 point more valuable than the compared column/row per match. Table 9 shows all the comparisons completed with an SESOI of 1.0 for the columns of the pitch. Table 10 outlines the subsequent columns used in EPV-19 and the metres included on the pitch. Table 11 provides the comparisons completed for all rows on the pitch against an SESOI of 1.0. Table 12 provides the final rows used in EPV-19 and the metres included on the pitch.

| **Table 9:** Statistical comparisons between columns at an SESOI of 1.0. Where numbers are separated by commas, they have been averaged. Difference and significance provide the mean difference (95% confidence intervals) between the columns and whether this was significant according to an SESOI of 1.0. Combine? Indicates whether the column values were averaged before comparing to the next column. Comparisons began at the outermost columns before progressing more centrally. | | | | |
| --- | --- | --- | --- | --- |
| **Columns included** | **Difference** | | **Significance** | **Combine?** |
| 2 - 1 | 1.36 (1.06 – 1.66) | | *P*  = 0.0096 | No |
| 3 - 2 | 2.35 (2.06 – 2.63) | | *P*  < 0.0001 | No |
| 4 - 3 | 0.91 (0.62 – 1.18) | | *P*  = 0.7441 | Yes |
| 5 - 3,4 | 2.00 (1.75 – 2.24) | | *P*  < 0.0001 | No |
| 6 - 5 | 1.72 (1.44 – 2.00) | | *P*  < 0.0001 | No |
| 7 - 6 | 3.38 (3.09 – 3.66) | | *P*  < 0.0001 | No |
| **Table 10:** Final columns for EPV-19 and the average column match EPV obtained per fixture across the 2019 Super League season | | | | |
| **Column**  **(metres included)** | | **Column Match EPV per fixture (95% Confidence Intervals)** | | |
| 1 (0-3, 64-68m) | | 1.27 (0.97 – 1.57) | | |
| 2 (4-8, 59-63m) | | 2.63 (2.35 – 2.92) | | |
| 3 (9-18, 49-58m) | | 5.43 (5.18 – 5.68) | | |
| 4 (19-23, 44-48m) | | 7.43 (7.14 – 7.71) | | |
| 5 (24-28, 39-43m) | | 9.15 (8.87 – 9.43) | | |
| 6 (29-38m) | | 12.52 (12.24 – 12.81) | | |
| **N.B.:** Column metres are inclusive and measured from the left side of the pitch based on the journal figures. EPV values are reported in points. | | | | |

| **Table 11:** Statistical comparisons between rows at an SESOI of 1.0. Where numbers are separated by commas, they have been averaged. Difference and significance provide the mean difference (95% confidence intervals) between the rows and whether this was significant according to an SESOI of 1.0. Combine? Indicates whether the row values were averaged before comparing to the next row. Comparisons began inside the attacking teams try area before progressing up the pitch to the opposition try area. | | | |
| --- | --- | --- | --- |
| **Rows included** | **Difference** | **Significance** | **Combine?** |
| 2 - 1 | 1.30 (0.93 – 1.67) | *P*  = 0.0559 | Yes |
| 3 - 1,2 | 2.29 (2.04 – 2.53) | *P*  < 0.0001 | No |
| 4 - 3 | -0.07 (-0.30 – 0.16) | *P*  = 1.000 | Yes |
| 5 - 3,4 | -0.28 (-0.48 – -0.08) | *P*  = 1.000 | Yes |
| 6 - 3,4,5 | 0.07 (-0.12 – 0.26) | *P*  = 1.000 | Yes |
| 7 - 3,4,5,6 | 0.73 (0.54 – 0.91) | *P*  = 0.9983 | Yes |
| 8 - 3,4,5,6,7 | 0.56 (0.38 – 0.74) | *P* = 1.000 | Yes |
| 9 - 3,4,5,6,7,8 | 1.72 (1.54 – 1.89) | *P* < 0.0001 | No |
| 10 - 9 | 2.82 (2.59 – 3.05) | *P* < 0.0001 | No |
| 11-10 | 1.06 (0.82 – 1.29) | *P* = 0.3333 | Yes |

| **Table 12:** Final rows for EPV-19 and the average row match EPV obtained per fixture across the 2019 Super League season | |
| --- | --- |
| **Row**  **(metres included)** | **Total Row EPV per fixture (95% Confidence Intervals)** |
| 1 (-10 to 10m) | 0.97 (0.75 – 1.19) |
| 2 (11m to 70m) | 3.39 (3.25 – 3.53) |
| 3 (71m to 80m) | 5.10 (4.90 – 5.30) |
| 4 (81m to 100m) | 8.45 (8.28 – 8.61) |
| **N.B.:** Row metres are inclusive and measured from team in possession’s try line (0m). EPV values are reported in points. | |

After obtaining the columns and rows, a 24 zone model was created. However, due to the significantly reduced number of visits to all zones in row 1, it was decided to average across them all, forming a single zone for row 1. This zone covered the whole area from -10m to 10m. The resulting EPV-19 is shown in Figure 2.

Having identified the zones based on statistical analysis of their match EPVs, the zone values were calculated by taking the weighted average of all the EPV-308 zones that they covered. For example, zone 1’s value was the weighted average of the 56 EPV-308 zones it covered. See Figure 1 in the manuscript for more details.

**Figure 2: EPV-19. Left: Blank zones; Right: zone values, coloured as per Figure 2 in manuscript.**


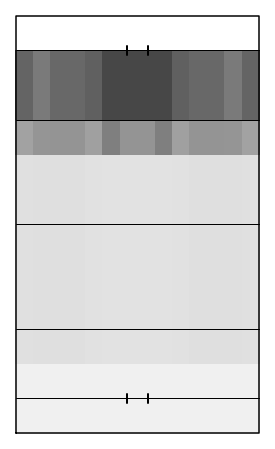

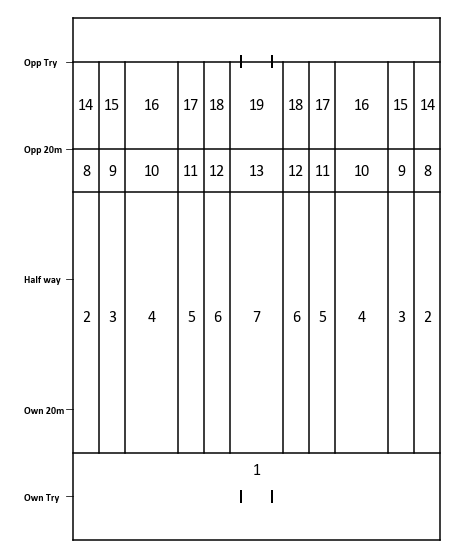


*EPV-13 (SESOI = 1.5)*

An SESOI of 1.5 indicates that we are testing whether across 2 matches, a column/row would be at least 3 points more valuable than the compared column/row. Table 13 shows all the comparisons completed with an SESOI of 1.5 for the columns of the pitch. Table 14 outlines the subsequent columns used in EPV-13 and the metres included on the pitch. Table 15 provides the comparisons completed for all rows on the pitch against an SESOI of 1.5. Table 16 provides the final rows used in EPV-13 and the metres included on the pitch.

| **Table 13:** Statistical comparisons between columns at an SESOI of 1.5. Where numbers are separated by commas, they have been averaged. Difference and significance provide the mean difference (95% confidence intervals) between the columns and whether this was significant according to an SESOI of 1.5. Combine? Indicates whether the column values were averaged before comparing to the next column. Comparisons began at the outermost columns before progressing more centrally. | | | | |
| --- | --- | --- | --- | --- |
| **Columns included** | **Difference** | | **Significance** | **Combine?** |
| 2 - 1 | 1.36 (1.06 – 1.66) | | *P*  = 0.8165 | Yes |
| 3 - 1,2 | 3.03 (2.78 – 3.28) | | *P*  < 0.0001 | No |
| 4 - 3 | 0.91 (0.62 – 1.18) | | *P*  = 1.000 | Yes |
| 5 - 3,4 | 2.00 (1.75 – 2.24) | | *P*  < 0.0001 | No |
| 6 - 5 | 1.72 (1.44 – 2.00) | | *P = 0.0614* | Yes |
| 7 – 5,6 | 4.24 (4.00 – 4.48) | | *P*  < 0.0001 | No |
| **Table 14:** Final columns for EPV-13 and the average column match EPV obtained per fixture across the 2019 Super League season | | | | |
| **Column**  **(metres included)** | | **Column Match EPV per fixture (95% Confidence Intervals)** | | |
| 1 (0-8, 59-68m) | | 1.95 (1.70 – 2.20) | | |
| 2 (9-18, 49-58m) | | 5.43 (5.18 – 5.68) | | |
| 3 (19-28, 39-48m) | | 8.29 (8.04 – 8.54) | | |
| 4 (29-38m) | | 12.52 (12.24 – 12.81) | | |
| **N.B.:** Column metres are inclusive and measured from the left side of the pitch based on the journal figures. EPV values are reported in points. | | | | |

| **Table 15:** Statistical comparisons between rows at an SESOI of 1.5. Where numbers are separated by commas, they have been averaged. Difference and significance provide the mean difference (95% confidence intervals) between the rows and whether this was significant according to an SESOI of 1.5. Combine? Indicates whether the row values were averaged before comparing to the next row. Comparisons began inside the attacking teams try area before progressing up the pitch to the opposition try area. | | | |
| --- | --- | --- | --- |
| **Rows included** | **Difference** | **Significance** | **Combine?** |
| 2 - 1 | 1.30 (0.93 – 1.67) | *P = 0.8581* | Yes |
| 3 - 1,2 | 2.29 (2.04 – 2.53) | *P*  < 0.0001 | No |
| 4 - 3 | -0.07 (-0.30 – 0.16) | *P*  = 1.000 | Yes |
| 5 - 3,4 | -0.28 (-0.48 – -0.08) | *P*  = 1.000 | Yes |
| 6 - 3,4,5 | 0.07 (-0.12 – 0.26) | *P*  = 1.000 | Yes |
| 7 - 3,4,5,6 | 0.73 (0.54 – 0.91) | *P*  = 1.000 | Yes |
| 8 - 3,4,5,6,7 | 0.56 (0.38 – 0.74) | *P* = 1.000 | Yes |
| 9 - 3,4,5,6,7,8 | 1.72 (1.54 – 1.89) | *P* = 0.0086 | No |
| 10 - 9 | 2.82 (2.59 – 3.05) | *P* < 0.0001 | No |
| 11-10 | 1.06 (0.82 – 1.29) | *P* = 0.9999 | Yes |

| **Table 16:** Final rows for EPV-13 and the average row match EPV obtained per fixture across the 2019 Super League season | |
| --- | --- |
| **Row**  **(metres included)** | **Total Row EPV per fixture (95% Confidence Intervals)** |
| 1 (-10 to 10m) | 0.97 (0.75 – 1.19) |
| 2 (11m to 70m) | 3.39 (3.25 – 3.53) |
| 3 (71m to 80m) | 5.10 (4.90 – 5.30) |
| 4 (81m to 100m) | 8.45 (8.28 – 8.61) |
| **N.B.:** Row metres are inclusive and measured from team in possession’s try line (0m). EPV values are reported in points. | |

After obtaining the columns and rows, a 16 zone model was created. However, due to the significantly reduced number of visits to all zones in row 1, it was decided to average across them all, forming a single zone for row 1. This zone covered the whole area from -10m to 10m. The resulting EPV-13 is shown in Figure 3.

Having identified the zones based on statistical analysis of their match EPVs, the zone values were calculated by taking the weighted average of all the EPV-308 zones that they covered. For example, zone 1’s value was the weighted average of the 56 EPV-308 zones it covered. See Figure 1 in the manuscript for more details.


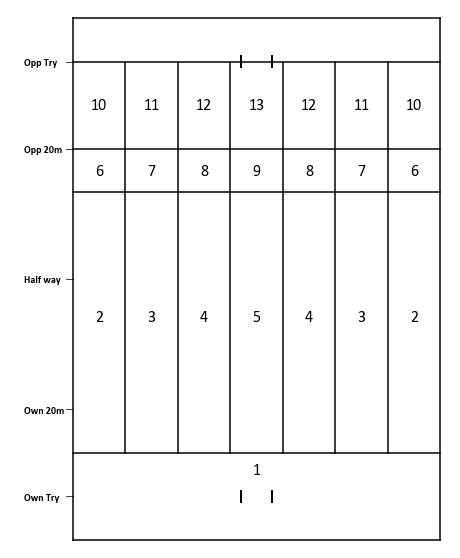

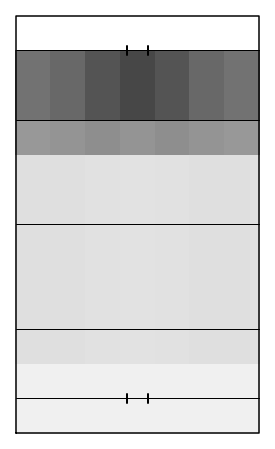
**Figure 3: EPV-13. Left: Blank zones; Right: zone values, coloured as per Figure 2 in manuscript.**

*EPV-9 (SESOI = 2.0)*

An SESOI of 2.0 indicates that we are testing whether across a single match, a column/row would be at least 2 point more valuable than the compared column/row. Table 17 shows all the comparisons completed with an SESOI of 2.0 for the columns of the pitch. Table 18 outlines the subsequent columns used in EPV-9 and the metres included on the pitch. Table 19 provides the comparisons completed for all rows on the pitch against an SESOI of 2.0. Table 20 provides the final rows used in EPV-9 and the metres included on the pitch.

| **Table 17:** Statistical comparisons between columns at an SESOI of 2.0. Where numbers are separated by commas, they have been averaged. Difference and significance provide the mean difference (95% confidence intervals) between the columns and whether this was significant according to an SESOI of 2.0. Combine? Indicates whether the column values were averaged before comparing to the next column. Comparisons began at the outermost columns before progressing more centrally. | | | | |
| --- | --- | --- | --- | --- |
| **Columns included** | **Difference** | | **Significance** | **Combine?** |
| 2 - 1 | 1.36 (1.06 – 1.66) | | *P*  = 1.000 | Yes |
| 3 - 1,2 | 3.03 (2.78 – 3.28) | | *P*  < 0.0001 | No |
| 4 - 3 | 0.91 (0.62 – 1.18 | | *P*  = 1.000 | No |
| 5 - 3,4 | 1.99 (1.75 – 2.24) | | *P*  = 0.518 | Yes |
| 6 - 3,4,5 | 3.06 (2.83 – 3.29) | | *P*  < 0.0001 | No |
| 7 - 6 | 3.38 (3.09 – 3.66) | | *P*  < 0.0001 | No |
| **Table 18:** Final columns for EPV-9 and the average column match EPV obtained per fixture across the 2019 Super League season | | | | |
| **Column**  **(metres included)** | | **Column Match EPV per fixture (95% Confidence Intervals)** | | |
| 1 (0-8, 59-68m) | | 1.95 (1.70 – 2.20) | | |
| 2 (9-23, 44-58m) | | 5.90 (5.67 – 6.13) | | |
| 3 (24-28, 39-43m) | | 9.15 (8.87 – 9.43) | | |
| 4 (29-38m) | | 12.52 (12.24 – 12.81) | | |
| **N.B.:** Column metres are inclusive and measured from the left side of the pitch based on the journal figures. EPV values are reported in points. | | | | |

| **Table 19:** Statistical comparisons between rows at an SESOI of 2.0. Where numbers are separated by commas, they have been averaged. Difference and significance provide the mean difference (95% confidence intervals) between the rows and whether this was significant according to an SESOI of 2.0. Combine? Indicates whether the row values were averaged before comparing to the next row. Comparisons began inside the attacking teams try area before progressing up the pitch to the opposition try area. | | | |
| --- | --- | --- | --- |
| **Rows included** | **Difference** | **Significance** | **Combine?** |
| 2 - 1 | 1.30 (0.93 – 1.67) | *P*  = 0.9999 | Yes |
| 3 - 1,2 | 2.29 (2.04 – 2.53) | *P*  = 0.0112 | No |
| 4 - 3 | -0.07 (-0.30 – 0.16) | *P*  = 1.000 | Yes |
| 5 - 3,4 | -0.28 (-0.48 – -0.08) | *P*  = 1.000 | Yes |
| 6 - 3,4,5 | 0.07 (-0.12 – 0.26) | *P*  = 1.000 | Yes |
| 7 - 3,4,5,6 | 0.73 (0.54 – 0.91) | *P*  = 1.000 | Yes |
| 8 - 3,4,5,6,7 | 0.56 (0.38 – 0.74) | *P* = 1.000 | Yes |
| 9 - 3,4,5,6,7,8 | 1.72 (1.54 – 1.89) | *P = 0.9991* | Yes |
| 10 - 3,4,5,6,7,8,9 | 4.31 (4.13 – 4.48) | *P* < 0.0001 | No |
| 11-10 | 1.06 (0.82 – 1.29) | *P = 1.000* | Yes |

| **Table 20:** Final rows for EPV-9 and the average row match EPV obtained per fixture across the 2019 Super League season | |
| --- | --- |
| **Row**  **(metres included)** | **Total Row EPV per fixture (95% Confidence Intervals)** |
| 1 (-10 to 10m) | 0.97 (0.75 – 1.19) |
| 2 (11m to 80m) | 3.61 (3.47 – 3.75) |
| 3 (81m to 100m) | 8.45 (8.28 – 8.61) |
| **N.B.:** Row metres are inclusive and measured from team in possession’s try line (0m). EPV values are reported in points. | |

After obtaining the columns and rows, a 12 zone model was created. However, due to the significantly reduced number of visits to all zones in row 1, it was decided to average across them all, forming a single zone for row 1. This zone covered the whole area from -10m to 10m. The resulting EPV-9 is shown in Figure 4.

Having identified the zones based on statistical analysis of their match EPVs, the zone values were calculated by taking the weighted average of all the EPV-308 zones that they covered. For example, zone 1’s value was the weighted average of the 56 EPV-308 zones it covered. See Figure 1 in the manuscript for more details.

**Figure 4: EPV-9. Left: Blank zones; Right: zone values, coloured as per Figure 2 in manuscript.**


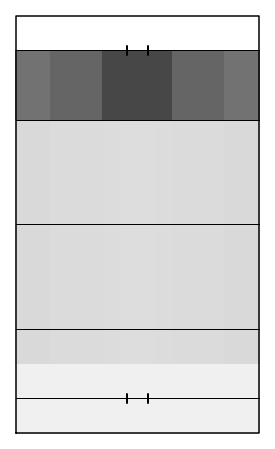

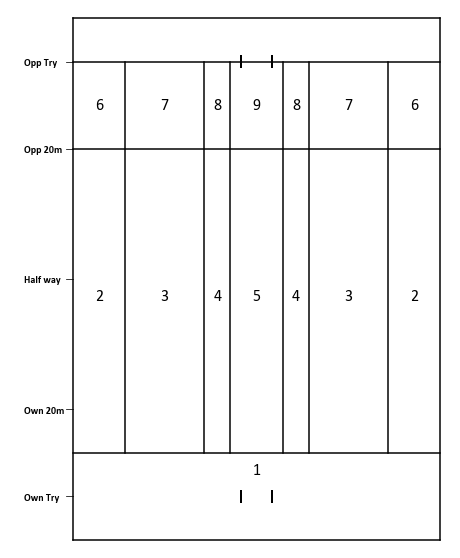

Supplement: S1 File — (DOCX) [file pone.0259536.s001.docx]
