# Supplementary material for: Development of an expected possession value model to analyse team attacking performances in rugby league
Source: PLoS One. 2021 Nov 12;16(11):e0259536. doi: 10.1371/journal.pone.0259536 (PMC8589207; doi:10.1371/journal.pone.0259536)
Supplement: Attachment — Submitted filename: Reviewer response.docx [file pone.0259536.s003.docx]

Reviewer #1: Thanks for your response and revising the manuscript.
I consider that the manuscript is ready for publication if the authors revise the following points.

*Thank you again for your critique during the review period. We are pleased that our changes satisfied your comments.*

**1. L167: EPV-37, EPV-19, EPV-13 and EPV-9, EPV-308 zones -> EPV-37, EPV-19, EPV-13, EPV-9, and EPV-308?**

*This sentence has now been clarified.*

“To calculate the aggregated set of zones for EPV-37, EPV-19, EPV-13 and EPV-9, the zones from EPV-308 were grouped together or split based upon differences in their match EPV.”

**2. KL divergence formula should be clarified (P and Q are not defined in this paper).**

*We have now added the KL Divergence formula to the text, with more description to ensure there is no ambiguity between P and Q, our true and approximating distributions.*

“The KL Divergence is calculated according to the equation:

$$D_{KL}(P|\left| Q \right)= \sum_{s\in S} P\left( s \right)\log\left( \frac{P(s)}{Q(s)} \right)$$

Where P is the true reward distribution, Q is the approximating reward distribution and S refers to the set of all states within the model. The subsequent match’s reward distribution (PG_mi_(S)) was used as the true reward distribution with the previous matches’ reward distribution (PG_M_(S)) as the approximating distribution. “
